# Molecular Dynamics Study of the Conformation, Ion Adsorption, Diffusion, and Water Structure of Soluble Polymers in Saline Solutions

**DOI:** 10.3390/polym13203550

**Published:** 2021-10-14

**Authors:** Gonzalo R. Quezada, Norman Toro, Jorge Saavedra, Pedro Robles, Iván Salazar, Alessandro Navarra, Ricardo I. Jeldres

**Affiliations:** 1 Departamento de Ingeniería Química, Universidad de Concepción, Concepción 4030000, Chile; 2 Faculty of Engineering and Architecture, Universidad Arturo Prat, Iquique 1100000, Chile; ntoro@ucn.cl; 3 Department of Wood Engineering, Universidad del Bío-Bío, Av. Collao 1202, Concepción 4030000, Chile; jsaavedra@ubiobio.cl; 4 Escuela de Ingeniería Química, Pontificia Universidad Católica de Valparaíso, Valparaíso 2340000, Chile; pedro.robles@pucv.cl; 5 Departamento de Ingeniería Civil, Universidad Católica del Norte, Antofagasta 1270709, Chile; isalazar@ucn.cl; 6 Department of Mining and Materials Engineering, McGill University, 3610 University Street, Montreal, QC H3A 0C5, Canada; alessandro.navarra@mcgill.ca; 7 Departamento de Ingeniería Química y Procesos de Minerales, Facultad de Ingeniería, Universidad de Antofagasta, Av. Angamos 601, Antofagasta 1240000, Chile; ricardo.jeldres@uantof.cl

**Keywords:** soluble polymers, flocculation, salinity, molecular dynamic, ion adsorption

## Abstract

Polymers have interesting physicochemical characteristics such as charge density, functionalities, and molecular weight. Such attributes are of great importance for use in industrial purposes. Understanding how these characteristics are affected is still complex, but with the help of molecular dynamics (MD) and quantum calculations (QM), it is possible to understand the behavior of polymers at the molecular level with great consistency. This study was applied to polymers derived from polyacrylamide (PAM) due to its great use in various industries. The polymers studied include hydrolyzed polyacrylamide (HPAM), poly (2-acrylamido-2-methylpropanesulfonate) (PAMPS), polyacrylic acid (PAA), polyethylene oxide polymer (PEO), and guar gum polysaccharide (GUAR). Each one has different attributes, which help in understanding the effects on the polymer and the medium in which it is applied along a broad spectrum. The results include the conformation, diffusion, ion condensation, the structure of the water around the polymer, and interatomic polymer interactions. Such characteristics are important to selecting a polymer depending on the environment in which it is found and its purpose. The effect caused by salinity is particular to each polymer, where polymers with an explicit charge or polyelectrolytes are more susceptible to changes due to salinity, increasing their coiling and reducing their mobility in solution. This naturally reduces its ability to form polymeric bridges due to having a polymer with a smaller gyration radius. In contrast, neutral polymers are less affected in their structure, making them favorable in media with high ionic charges.

## 1. Introduction

Soluble polymers can significantly improve solid–liquid separation processes and are increasingly used in various industries, including mineral recovery, papermaking, wastewater treatment, and dewatering of mining tailings [1,2,3]. Fundamentally, it is required that molecules can maintain an adequate extension in the medium to interact with several suspended particles forming large aggregates, which settle due to gravitational effects.

Of particular interest in this study is analyzing macromolecules that may potentially improve tailings management practices, where different variables influence this process, with the properties of the polymer being probably the most attractive. This is because the mineralogy and the liquid medium strongly influence the efficiency of the thickening processes, but the chances to control them are practically nil. Another relevant issue is technological aspects, but the thickening technologies are established when designing the plant. It is rare to make significant design changes as this involves high costs; laboratory-scale tests that can usefully anticipate the impact of design changes are not trivial and require specialized knowledge. In contrast, handling reagents is a fundamental activity in operations that offers excellent scope for manipulation since it is simple to switch conditions such as the type of flocculant, dosages, or injection points.

The wide variety of commercial polymers that are available on the market can already generate distinct behavior. For example, Grabsch et al. [4] studied the flocculation kinetics of two commercial polyacrylamide (PAM)-based flocculants, Rheomax^®^ DR 1050 and BASF Magnafloc^®^ 336, when applied to a suspension of fine calcite, confirming very different responses to variations in the concentration of solids. The conventional Magnafloc 336 acrylamide/acrylate copolymer was superior at lower solid levels. Both products gave a comparable performance for low dosages at higher solid levels, conditions in which the effective aggregate volume fraction does not significantly impact the aggregate sizes achieved. However, Rheomax DR 1050 consistently produced larger aggregate sizes and better sedimentation rates for higher dosages at solid concentrations of ≥ 80 kg/m^3^, consistent with a denser aggregate structure. Tanguay et al. [5] subsequently used these kinetic results to model in 3D the potential consequences on feedwell performance, predicting the scope for doubling solids throughput under some conditions by merely changing the type of reagent.

The distinctions in the performance described above originated from the different physicochemical characteristics of the polymers, such as charge density, branching, chemical functional group, and molecular weight. Achieving a comprehensive understanding of the impact of these properties is complex, but the efforts of numerous groups have provided valuable insights on the interaction of polyacrylamide-based polymers with solid particles. For example, Costine et al. [6] studied the flocculation performance of kaolin suspensions, analyzing the influence of mixing intensity, solids concentration, and liquid conditions (e.g., pH, salinity) on the response of seven anionic PAMs at a fixed anionic charge but varying molecular weight (MW). Under gentle mixing, lower MWs gave a more effective dosage response, producing denser aggregates and settling faster than equivalent sizes produced with higher MWs. In contrast, the larger sizes created by the long chains gave access to faster sedimentation rates under intense mixing. Yousefi et al. studied the influence of the type and structure of polyelectrolytes on the rheological [7], electrokinetic, and dewatering characteristics [8] of industrial sludges from a highly stable membrane bioreactor (MBR). Several similar studies have analyzed the effect of PAM-based polymer properties on particle flocculation under conditions of relevance to the oil industry, wastewater treatment, and papermaking [9,10,11,12,13,14]. Unfortunately, the knowledge achieved to date is not enough to allow operators to develop flocculant management criteria based on understanding the reagent’s chemistry.

The industry presents increasing challenges such as the processing of low-grade minerals, water scarcity, regulations that require substantial improvements in the extraction of water from tailings, the presence of complex gangue (e.g., clays), or the use of low-quality water (e.g., seawater). Unfortunately, the traditional polyacrylamide monomer base does not always provide the desired outcomes due to functional groups’ varying physicochemical properties or limitations. For example, PAM is highly hydrophilic and forms loosely packed aggregates that retain a significant water content, making it challenging to remove thickened tailings, especially when they are deposited [15]. The standard free-radical polymerization method is commonly used to produce these reagents, but the properties of the generic products can display broad distributions, especially in molecular weight [16]. Traditional flocculants limit their extension in saline medium, especially when divalent ions such as calcium and/or magnesium are present [6,17]. The strong electrostatic compression caused by these cations reduces the repulsion between the anionic functional groups, reducing the volume of the polymer in solution, limiting its ability to form polymeric bridges. In this context, molecular dynamics (MD) studies have made it possible to improve the understanding of interactions at the molecular scale between polyelectrolytes and minerals’ surfaces. For example, Quezada et al. [18] directly observed how a saline medium altered the conformation of an hydrolyzed polyacrylamide (HPAM) in solution and its adsorption on the quartz surface. Even the authors later studied the types of bonds generated by the interaction of HPAM on quartz, kaolin, and brucite surfaces [19]. The latter is one of the main seawater precipitates that are generated by bringing the pH to highly alkaline conditions than impair flocculation. The MD results explained that the adsorption is mainly carried out by the interaction between the deprotonated oxygen from the acrylic group of the polymer and the oxygen from the hydroxide of the brucite surface. There is also a significant contribution of hydrogen bonding between nitrogen from the acrylamide group and oxygen from the hydroxide.

In general, traditional anionic polyacrylamides reduce their efficiency in highly saline environments. Therefore, it is attractive to look for new soluble polymers that preserve their extension in solution, even in low-quality water, which could have significant benefits on mineral flocculation, such as quartz and clays. In this context, PAM derivatives such as poly (2-acrylamido-2-methylpropanesulfonate) polyelectrolyte (PAMPS), have been used in extreme saline brines as a stabilizer in silica nanoparticle coating due to the synergistic action between strong sulfonate groups and hydrophilic amide groups [20,21] and anticoagulant applications [22].

Polyacrylic acid (PAA) can adsorb large amounts of liquid, which is why it is used as an adsorbent compound [23] and as a nanocomposite [24]. Studies about the conformation of PAA show that the presence of saline electrolytes tightly coils the polymer [25,26], but the critical concentration at which coiling begins is unknown. Recently developed studies showed a good ability to adhere to clay surfaces, even in saline media such as seawater [27]. Quezada et al. [28] identified that the main interaction by which the polymer is adsorbed is through the hydroxyl of the mineral surface and the COO^−^Na^+^ complexes. Mpofu et al. [29] analyzed the effect of the polymer structure on the flocculation of kaolinite suspensions. Nonionic polyethylene oxide (PEO) had a higher affinity to the surface when compared with anionic polyacrylamide. Its neutral behavior and its oxygen backbone can have an interesting behavior in the presence of saline ions, and in addition, its adopted conformation is mostly extended, as was later stated by McFarlane et al. [30].

Another interesting polymer is the guar gum (GUAR), which has a wide range of applications from the textile to the food industries [31]. Some researchers have explored its potential use in the mining industry. For example, Castellón et al. [32] showed that this polysaccharide could function as a pyrite depressant in seawater, or research has even been carried out to analyze its adsorption on quartz surfaces in highly saline media. Ma and Pawlik [33] studied the effect of different monovalent cations, proposing that this polymer competes with water to access the silanol sites on the quartz surface.

This represents a new research opportunity, which is addressed in the present research, aiming to improve the understanding of conformation, counterion condensation, and water structure of polymers in saline solutions. In this work, computational simulation is used to study the effect of aqueous solutions of NaCl dissolved with six different polymers: HPAM, PAA, PAM, PAMPS, PEO, and GUAR. The study focuses on measuring the interaction of the polymer with salt ions, the conformation of polymers, and the affinity of water. The objective is to characterize polymers and to show their potential use in highly saline systems based on the principles of MD and QM that allow the polymers to be rigorously modeled.

## 2. Methodology

### 2.1. Polymers

**Figure 1:**
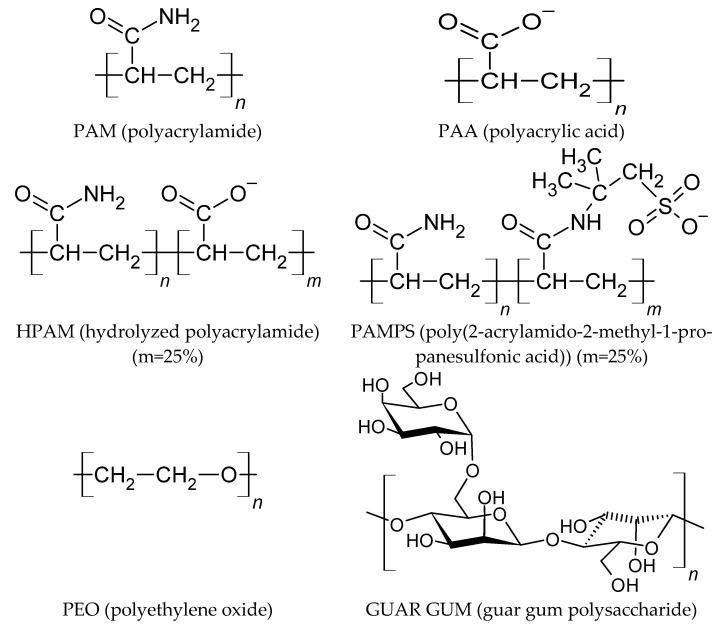
Monomer structure of the molecules studied in this work.

The structures of these polymers are outlined in Figure 1. The first base polymer is neutral polyacrylamide (PAM); if a hydroxide replaces the amine group, it is possible to obtain polyacrylic acid (PAA), which at pH over 4.5 is found mostly dissociated. Likewise, it is possible to obtain copolymers of PAM together with PAA, which is known as hydrolyzed polyacrylamide (HPAM). The most used has 30% of its monomers as the PAA-type; it was set at 25% for simplicity in this work. It is also possible to generate a PAA copolymer with a 2-acrylamide-2-methyl-1-propane sulfonic acid (AMPS) monomer. In this case, it was also set at a percentage of 25% for comparison with HPAM. At pH conditions above 7, the sulfonate group is also dissociated. Then, two polymers that are not derived from PAM were studied, polyethylene oxide (PEO), a linear chain without hanging groups, and guar gum polysaccharide (GUAR), which consists of a β-D-mannopyranose and an α-D-galactopyranose attached to a manopyranose. Hydrogen was chosen as the end group for the polymers derived from PAM. In the case of PEO and GUAR polymers, the H or OH groups were used to terminate the polymers. The chosen configuration of the monomers in this work was syndiotactic because it is a homogeneous structure.

### 2.2. Forcefield

In this work, we chose to use the Generalized Amber Force Field (GAFF) because it successfully simulates polymers in solution [28,34,35]. To do this, the Antechamber program [36,37] was used first to generate the topology of the polymers to be studied; this includes the van der Waals potentials, bonding, angles, and dihedrals. The polymers were constructed with at least three repeating monomers to consider all of the interactions in both the terminal and central monomers. Then, we proceeded to determine the partial charges of the polymers using the methodology of the restricted electrostatic potential (RESP) [38]. This calculation was made using the R.E.D. III.52 [39] in conjunction with the Gaussian program [40]. The PAM, PEO, and GUAR polymers are neutral at pH 7, while the HPAM, PAA, and PAMPS polymers have a negative charge depending on the number of monomers. To parameterize the polymers, the sizes of 12 monomers were used and their charges were adjusted with the equation:

(1) $$q_{i}=q_{i0}-\frac{\sum q_{i0}-Q_{tg}}{N}$$

where $q_{i0}$ is the charge obtained by the RESP methodology, $Q_{tg}$ is the target charge of the monomer, and *N* is the number of atoms of the monomer. For the PAM, PEO, and GUAR polymers, $Q_{tg}$ was set equal to zero. In HPM, PAMPS, and PAA polymers, the value of $Q_{tg}$ was equal to −1 for charged monomers and 0 for neutral monomers. All partial charges and forcefield parameters for the polymers are provided in the Supplementary Materials. In the case of the dissolved Na^+^ and Cl^−^ ions, the non-bonding parameters used were correctly adjusted for the ion-oxygen distance from the work of Li et al. [41] for monovalent ions. For water, the SPC/E water model [42] was used in conjunction with the SETTLE constraints for the molecule’s geometry [43].

### 2.3. Initial Configuration

An original code generated the initial configuration. First, the polymer of the desired size is generated; in HPAM, PAM, PAMPS, and PAA, 48 monomers were used, 32 were used for PEO, and 12 were used for GUAR. The configuration was carried out syndiotactically between the pendant monomers. The number of monomers chosen was related to the length of the main chain, which was similar for each polymer; for polymers derived from PAM, its initial length was 11.8 nm; for PEO, it was 11.5 nm; and for GUAR was 12.5 nm. The HPAM and PAMPS copolymers with 25% substitutions have 36 AM monomers and 12 AA or AMPS charged groups. A 25% substitution in copolymers means that one of the AAs or AMPSs is replaced for every three acrylamide monomers. These polymers (except for GUAR) are added to a cubic box of an initial size of 10 nm with periodic edges in all three dimensions. This size is sufficient to avoid interaction with the periodic image of the polymer [34]. From inspection, in the case of GUAR, it was preferred to increase the initial size to 12 nm to avoid contact with its periodic image because it is a more voluminous polymer. Then, the dissolved ions were added: first, the counter ions of the polymer (in this case Na^+^) to neutralize its net charge, followed by dissolved salt such as NaCl, which is the most abundant in saline waters. The ions were added with the restriction of being separated from the 0.8 nm polymer. The concentrations studied were 0.006, 0.06, and 0.6 M. Then, pre-balanced water at 300 K was inserted into the box with the ions and the polymer. The water molecules were eliminated when they overlapped with the ions and the polymer. For this, a distance between atoms less than 0.2 nm was defined. The conditions of the systems studied have been summarized in Table 1.

### 2.4. Molecular Simulation

The behavior of a single polymer molecule under saline conditions was studied through molecular dynamics using the Gromacs simulation package version 2020.5 [44]. To use the force field derived through the antechamber, the ACPYPE program [45] transforms the obtained topology into the language of the Gromacs simulation program. The initial setup was relaxed in a force minimization step to decrease the probability of simulation crash. Then, the system was simulated in an NVT simulation for 0.1 ns at a temperature of 300 K, keeping the polymer and the fixed ions to form the hydration layers around them. Then, it was simulated in a collective NPT for 2 ns at a temperature of 300 K and a pressure of 1 bar for reference to relax the simulation box. Then, in an annealing NVT simulation for 10 ns where the temperature was raised from 300 to 450 K for 0.001 ns and kept at 450 K until 5 ns, it was slowly decreased from 450 to 300 for 5 ns. This simulation was performed to relax the polymer from unfavorable or metaestable configurations. Finally, a simulation is run for the production of data of 400 ns in the NVT collective at 300 K. We performed three simulations for each case to eliminate bias in the results. The integration step was 2 × 10^−6^ ns, which, together with the restrictions of the hydrogen bonds using LINCS [46], is suitable so that the simulation does not present artifacts or energy losses. Temperature and pressure controls were carried out by the Nose–Hoover thermostat [47,48] with a relaxation time of 0.0025 ns and by the isotropic Parrinello–Rahman barostat [49] with a relaxation time of 0.001 ns. The cutoff radii for the van der Waals and coulombic energies were equal to 1.2 nm. Long-range corrections were used by the Ewald particle mesh method [50]. The cross interactions in the LJ energy were defined with the Lorentz–Berthelot mixing rules.

## 3. Results

### 3.1. Radius of Gyration

**Figure 2:**
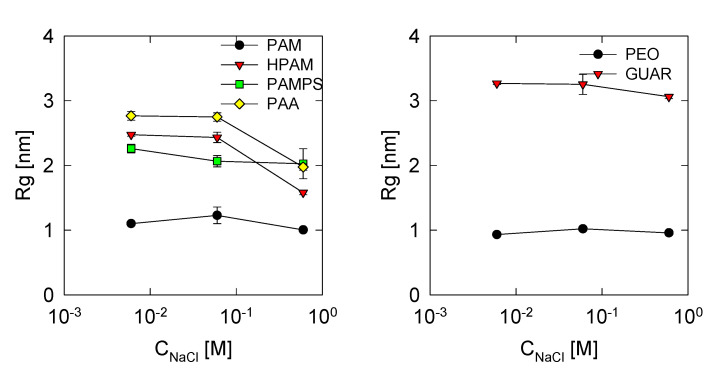
Radius of gyration of the polymers studied at different concentrations of NaCl.

The radius of gyration (Rg) directly indicates the degree of coiling that a molecule has by measuring the distance that the atoms of the molecule have with respect to its center of mass. This calculation was carried out only with the carbon atoms of the main chain, and the results are plotted in Figure 2.

Among the polymers derived from PAM, it is observed that this same polymer has a more coiled structure than the rest. When the concentration of salt increases, a slight increase in Rg is observed, but even so, it stays within a value of the radius of gyration of 1.1 nm. The PAM, being neutral, shows only attraction with itself mainly and therefore generates this behavior. In HPAM, it is observed that the radius of gyration increases to 2.5 nm. This shows that the charged acrylate groups (COO) greatly influence the stretch of the polymer; however, that also implies that it is more susceptible to the presence of salts. The results show that the radius of gyration decreases to a value of 1.5 nm when the salt concentration is 0.6 M. Such results are comparable with those shown by Chen et al. [51] for both PAM and HPAM. 

The PAMPS polymer exhibits a slightly smaller radius of gyration than HPAM. The increase in salinity did not significantly affect their coiling; therefore, it is inferred that the PAMPS charged groups are less strong than the COO, considering that the HPAM coiling was more intense. For PAA, it is possible to observe that it has a behavior similar to that of HPAM; evidently, the greater quantity of charged groups increased the radius to a value of 2.8 nm since the repulsion between charged groups increases. Likewise, the presence of salt generated a decrease in the radius of gyration to a value of 2.0 nm because the ions are adsorbed on stronger charged groups. The ion adsorption analysis is discussed in Section 3.2. For PEO and GUAR polymers, a behavior similar to that of PAM is observed because they are neutral polymers and the effects of salinity are less intense. GUAR shows the largest of the radii of gyrations because it is a large polymer in terms that it is thicker due to the cyclic groups of the saccharides. In the case of PEO, it shows a lower value than PAM because it is a linear polymer.

**Figure 3:**
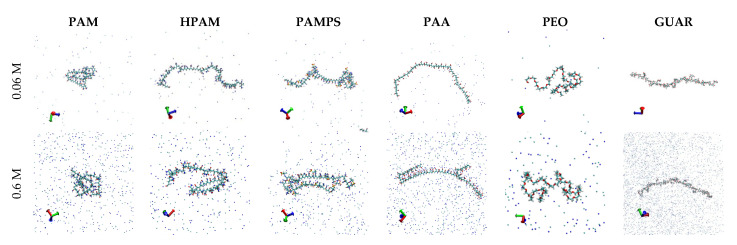
Snapshot of polymer configuration obtained from the simulation at 0.06 and 0.6 M for the six polymers studies.

To better understand the radii of gyration, the equilibrated configurations of the polymers studied have been obtained. In Figure 3, the configurations that are generated in the simulations for the cases of salt concentration 0.06 and 0.6 M have been placed. The conformation of the polymers can be observed directly from these images.

In the case of PAM, a highly coiled structure is observed in both salt concentrations, in accordance with the low radii of gyration regardless of the salt concentration. The HPAM shows that the presence of acrylate groups generates a stiffness in the chain but that, at high concentrations of salt, the chain manages to twist for the formation of internal bridges, and the latter is validated considering the decrease in 40% compared with the radius of gyration to 0.06 M NaCl. The PAMPS polymer shows similar behavior to HPAM, where at high concentration, part of the chain also twists to form bonds, but due to the larger size of the pendant group, it decreases less.

In the case of PAA, the degree of stretching is maximum (in accordance with the greater radii of gyration obtained among the studied polymers) due to the high repulsion of its charged groups, only at high concentrations, and the formation of cationic bridges allowing bending of the chain-forming cationic bridges. Since the PEO polymer has a low charge density, it coils with very low interaction with the medium, which is reflected in the small radii of gyration of the order of 1 nm and practically independent of the salt concentration. Finally, the GUAR presents the highest stretch of all its hanging groups and rings, allowing for a low rearrangement of the chain so that, even at high salt concentration, there are no appreciable changes in the structure. The latter also follows the high radii of gyration and is independent of the salt concentration.

### 3.2. Counterion Condensation

**Figure 4:**
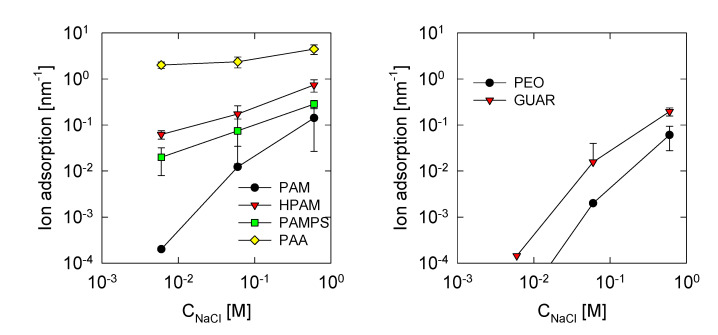
Na^+^ ion adsorption per unit length of the studied polymer. Error bars for adsorptions less than 10^−2^ were omitted.

To understand the results of the radius of gyration, it is necessary to know the linear adsorption of ions on polymers, known as ion condensation [52]. This was found by calculating the number of ions within a cutoff radius of less than 0.3 nm between the polymer atoms and the Na+ cations. The results presented in Figure 4 show that the adsorption of the ion increases with the salt concentration for all of the cases studied. This implies that, the higher the quantity of ions present, the higher the probability that these enter within the radius necessary to be adsorbed on the polymer.

In polymers derived from PAM, it is observed that ion adsorption follows the sequence PAA > HPAM > PAMPS > PAM. Clearly, the greater amount of PAA charged sites translates into greater Na+ adsorption with a value above 2 nm^−1^. In these cases, the oxygens of the COO groups have a value of −0.80e. Next in the sequence is HPAM, which shows adsorption between 0.1 to 1 nm^−1^. There are only 25% of acrylate monomers; therefore, in theory, it should decrease to 25% of PAA results. Figure 4 shows that, for 0.6 M, the adsorption for PAA is 52 nm^−1^, and 25% corresponds to 13 nm^−1^, but the adsorption for HPAM at 0.6 M is 8.7 nm^−1^. This shows a greater decrease than expected, indicating a group effect that depends on the adsorption of ions. For PAMPS, there is lower adsorption compared with HPAM, suggesting that the SO_3_ groups of PAMPS have a lower capacity to adsorb ions than the COO of the HPAM molecule. This is expected since the partial charge for SO_3_ oxygens equals −0.61e, and for COO, it is around −0.74e. The PAM polymer has the lowest adsorption because it is a neutral polymer that has the lowest affinity for the cations present. Even so, there is appreciable adsorption at concentrations of 0.6 M, similar to those of HPAM at the concentration of 0.06 M. This shows that carbonyl oxygens have an affinity with cations, where their partial charge is around −0.52e. In the case of PEO, we see the least adsorption: its neutrality and high coiling mean that a cation is less likely to be adsorbed on the main chain oxygens. Finally, the GUAR presents adsorption comparable with the PAM polymer, indicating that the hydroxide groups allow for the adsorption of cations.

It is important to mention that, under Manning’s criterion, only PAA should exhibit the ion condensation (Γ > 1). Still, as we can see in the simulation, all of the polymers studied have a degree of adsorption. Therefore, the Manning criterion helps to define if the polymer adsorbs appreciable amounts of ions.

### 3.3. Water Orientation

**Figure 5:**
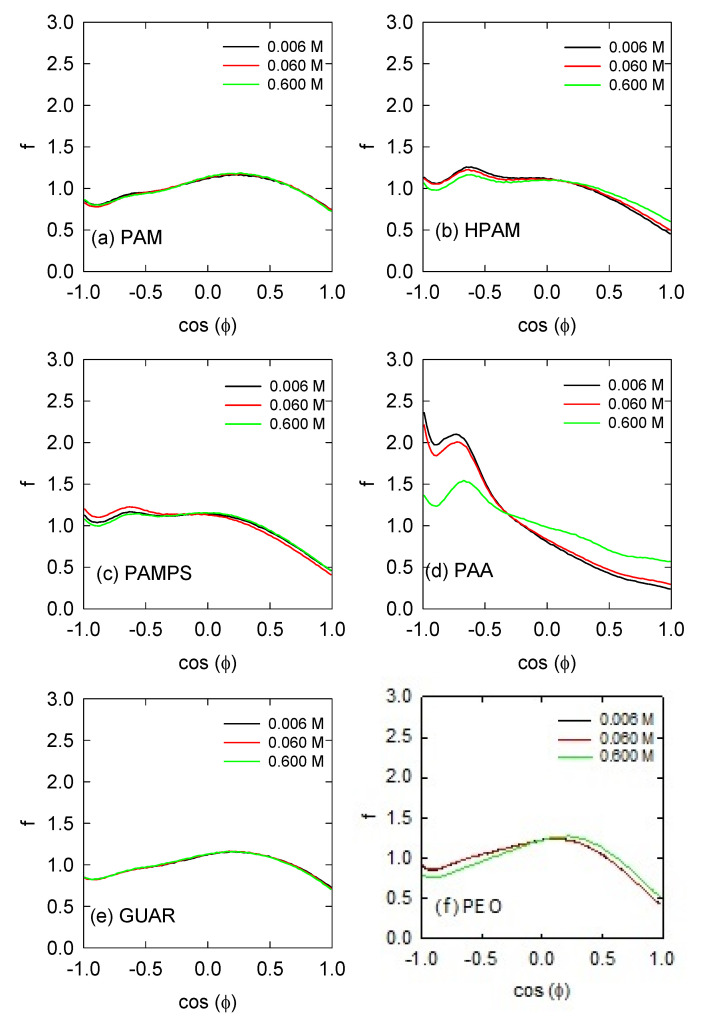
Orientation of water molecules around the polymers at different NaCl concentrations. (**a**) poly-acrylamide, (**b**) hydrolyzed polyacrylamide, (**c**) 2-acrylamido-2-methyl-1-propanesulfonic acid, (**d**) polyacrylic acid, (**e**) guar gum, (**f**) polyethylene oxide.

The polymers studied have different structures and electrical charges, so it is necessary to quantify the interaction with the aqueous medium. Depending on the interaction of the polymer with water, it is possible to describe the behaviors about the mobility of the molecule or the affinity of interacting with another molecule or mineral surface. This can be determined through the orientation of the water molecules on the polymers. The orientation of the water layers is determined as a frequency distribution of the angles that the water molecules adopt on average around all of the atoms of the polymers. In this case, it is carried out over a cut-off radius of 0.5 nm to include approximately the first layer and half of the second layer. These results were plotted in Figure 5 for all of the polymers studied. Negative cos (ϕ) values represent when the hydrogens are oriented towards the polymer atoms, while positive values are when the oxygens are oriented towards the polymer atoms.

The PAM polymer shows a homogeneous distribution where the highest frequency occurs around a value of +0.25, which means that a single hydrogen in the water molecule is slightly oriented towards the polymer. For the HPAM and PAMPS molecules, there is an increase in the frequency values of −0.6 and −1.0. This indicates that the orientation of the hydrogens is stronger because, at cos(ϕ) equal to −0.6, the hydrogen atom is in direct line with polymer atoms [53] while, at −1.0, both hydrogens are oriented to the atoms of the polymer. The ability to orient the water molecules of HPAM is greater than PAMPS because the SO_3_ groups are weaker than the COO. In the case of PAA, it clearly shows that it is a polymer with an electrically strong character and generates a high capacity to orient the water molecules in the peaks at −0.6 and −1.0. Due to this strong character, it is also susceptible to excess ions, decreasing the orientation of the water when salt concentrations are high. 

Finally, the PEO and GUAR polymers show behaviors similar to that observed in PAM, which is also neutral. That is, they show a peak in distribution at a value of +0.25. Interestingly, there is also a slight peak at −1 in the three polymers of PAM, PEO, and GUAR, indicating that water molecules interact with their two hydrogens towards the surface of the polymers. This may be due to the interaction of water with two oxygen groups; in the case of PAM, at two carbonyls of acrylamide; while for PEO and GUAR, with two ethers of the main chain [54].

In general, the effects of salinity are only present in PAA. This is directly related to the adsorption of ions and the tortuous chain that modify the interaction of the polymers with the water around them.

### 3.4. Diffusion Coefficient

**Figure 6:**
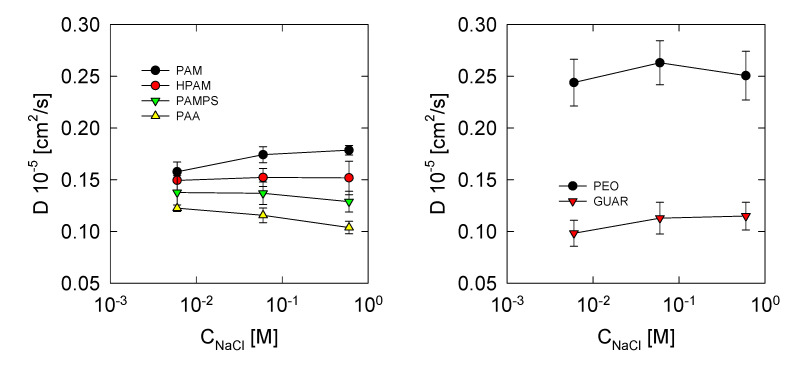
Diffusion coefficient of polymers at different salt concentrations at 300 K.

The diffusion coefficient allows for estimating the mobility and viscosity of polymers in water and for seeing the effects of salt. For this, the mean square displacements (msd) were determined, where 20,000 restarts were made, and the adjustment was carried out mainly in the interval from 1 ps to 10,000 ps, where the curves present their greatest linearity. This interval is above the ballistic regime of the molecule. The results are shown in Figure 6 for the systems studied.

It is observed that the diffusion coefficients for polymers derived from PAM present values lower than 0.2 × 10^−5^ cm^2^/s, which is ten times less than the diffusion coefficient of water [55]. The following sequence is generally observed in the diffusion coefficients PAM > HPAM > PAMPS > PAA. This scales directly with the electrical nature of the polymer. The PAM is neutral, so it does not have counter ions, and there is also no strong interaction with the environment, according to the results of Figure 5. Therefore, it presents less impediment in diffusion. In HPAM, the charged groups attract the counter ions and the water layers neighboring the molecule; this hinders its free movement in the medium. The PAMPS has the same charge density as the HPAM but a larger dangling group, which hinders its movement. PAA has a high linear charge, so a lower diffusion coefficient than the rest since is expected its hydration layer and ion adsorption hinder its mobility. PEO polymer has the highest diffusion coefficient. This effect is produced by the low adsorption of ions and low affinity with the medium, allowing for the highest diffusion. On the other hand, GUAR has the lowest diffusion coefficient because it is one of the polymers with the highest molecular weight and with low interaction with the medium. Interestingly, the effects of salt concentration affect each polymer differently. In the PAM, it is observed that, when the salinity increases, greater mobility of the polymer is generated; This may be because the interaction of PAM with the environment decreases with the presence of salt and allows for its free mobility. For HPAM, the effect of salt is minimal. PAMPS presents appreciable effects, and as salinity increases, the diffusion coefficient of the polymer decreases. This shows that dangling groups are more susceptible to the presence of salts. For PAA, the excess salt also causes a decrease in the diffusion coefficient, caused by the greater adsorption of ions. Similar to PAM, GUAR presents an increase in the diffusion coefficient due to the lower interaction with the medium. Finally, the PEO presents the highest values in the diffusion coefficient. A maximum value is observed at 0.06 M, and such a behavior may be because, at high concentrations, there is adsorption of ions (Figure 4), which could influence the mobility of the polymer.

### 3.5. Polymer Self-Interactions

To understand the results presented above, this section on polymer interactions has been included. Such interactions refer to hydrogen bonds and cationic bridges commonly found in polymers [19,28]. The cationic bridges are the product of the union between the cations with the negative atoms of the polymers; in this work, the following are considered: the oxygen of the carbonyl group in the acrylamide monomers (R-CONH_2_), the charged oxygen of the monomers of acrylate (R-CO_2_^−^), oxygen in the charged groups of the sulfonate group (R-SO_3_^−^), and ether oxygen (ROR) and the hydroxyl oxygen (R-OH) of the PEO and GUAR polymers. 

The formation of cationic bridges occurs when the sodium cation binds simultaneously with two of the negative atoms mentioned above. This is true when the distance is less than 0.3 nm between a cation and oxygen [28]. On the other hand, hydrogen bonds are also determined between electronegative groups but linked by a hydrogen atom that is covalently linked to one of the electronegative groups. Electronegative atoms include those already mentioned above, and amino groups are also included in acrylamide. The criterion, in this case, is a distance less than 0.30 nm between electronegative atoms [28].

**Figure 7:**
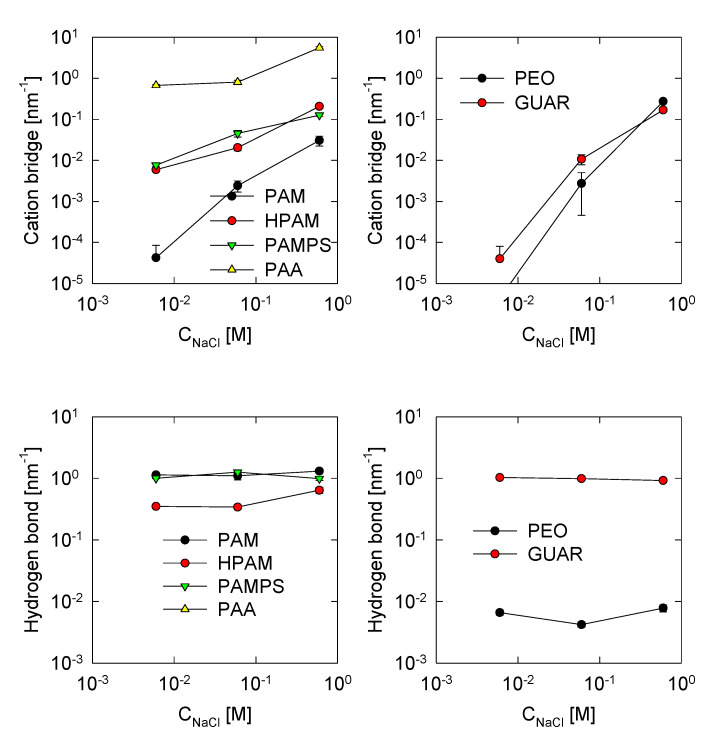
Polymer self-cation bridge and hydrogen bond interactions for all systems studies.

The results of this analysis are presented in Figure 7, where they were divided by the total length of the polymer, giving a linear density. 

First, the effect of salt is only observable for cationic bridges clearly because its presence is necessary for them to occur. The density of hydrogen bonds is practically not influenced by the concentrations of salts. We observe that the PAA polymer exhibits the highest cationic bridge formation. The conformation of PAA is only a function of cationic bridges since hydrogen bonds do not exist. Therefore, the HPAM polymer has fewer cationic bridges than PAA due to its fewer number of acrylate monomers. The hydrogen bond is greater than the cationic bridges, but the cationic bridges give the effects on the conformation of the polymer, which means they are stronger interactions.

Similar results to those of HPAM are found for the PAMPS polymer, in which the number of cationic bridges is similar even when the functional group is different. A remarkable fact about the hydrogen bonding behaviors between PAM, HPAM, and PAMPS is that HPAM has the least amount of hydrogen bonds. This is because HPAM has fewer sites to form hydrogen bonds than PAM and PAMPS, mainly due to the presence of amines (Figure 1). The PAM polymer has the fewest cationic bridges due to less sodium adsorption, and the amount of hydrogen bonds is similar to that of the PAMPS molecule because both have a similar number of groups capable of forming hydrogen bonds.

The PEO polymer has a similar tendency toward PAM except at high salt concentrations, where there are more cationic bridges with PEO. In the case of hydrogen bonds, they are low because few hydroxyl groups allow this bond. Finally, the GUAR polymer has a similar number of cationic bridges to PEO and PAM, and therefore, this type of interaction is rare. This polymer has many hydroxyl groups that can form hydrogen bonds; however, the amount obtained is similar to that of the PAM or PAMPS polymers, so the structure of GUAR does not allow for a high number of hydrogen bonds.

## 4. Conclusions

A molecular dynamics study was carried out to understand the interactions between various polymers and their aqueous environment, particularly analyzing the effect of salinity. These polymers, including PAM, HPAM, PAA, PAMPS, PEO, and GUAR, could have potential use in industrial processes when the process water has a high salt content. The results in the family of polymers derived from PAM show that adding charged groups substantially modifies the structure and mobility of the molecules. A considerable increase in the stretching of polymers is observed due to the repulsion between their charges, which favors their ability to form hydrogen bonds with solid particles suspended in the medium. This also alters the hydration layer of the polymer and its diffusion in the medium. In polymers of a different nature, such as PEO, the behavior is similar to PAM, with coiled conformation and greater mobility. In the case of GUAR, its monomers are larger, which inherently generates a more stretched polymer with a slower diffusion. However, the effect caused by salinity is particular to each polymer, observing that polymers with an explicit charge or polyelectrolytes are more susceptible to changes due to salinity, increasing their coiling and reducing their mobility in solution. This naturally reduces its ability to form polymeric bridges due to having a polymer with a smaller gyration radius. In contrast, neutral polymers are less affected in their structure, favorable in media with high ionic charges. This work has demonstrated the utility of MD and QM techniques in characterizing polymers of different nature and structures and obtaining valuable and consistent results.

## Figures and Tables

**Table 1:** Summary of the systems studied.

Polymer	Molecular Weight	Polymer Lenght	NaCl	Na^+^ Counterion	Na^+^ Salt Cation	Cl^−^ Salt Anion	Water Molecules
	[g/mol]	[nm]	[mol/L]				
PAM	3413.81	11.8	0.006	0	4	4	32,408
			0.06	0	36	36	32,326
			0.6	0	361	361	31,618
HPAM	3413.53	11.8	0.006	12	4	4	32,401
			0.06	12	36	36	32,316
			0.6	12	361	361	31,619
PAMPS	5035.69	11.8	0.006	12	4	4	32,339
			0.06	12	36	36	32,248
			0.6	12	361	361	31,550
PAA	3412.69	11.8	0.006	48	4	4	32,373
			0.06	48	36	36	32,319
			0.6	48	361	361	31,608
PEO	1427.72	11.5	0.006	0	4	4	32,499
			0.06	0	36	36	32,410
			0.6	0	361	361	31,715
GUAR	5855.16	12.5	0.006	0	4	4	32,314
			0.06	0	36	36	32,228
			0.6	0	361	361	31,483

PAM—polyacryl amide; HPAM—hydrolized polyacryl amide; PAMPS—poly(2-acrylamido-2-methyl-1-propanesulfonic acid); PAA—polyacrylic acid; PEO—polyethylene oxide; GUAR—guar gum polysaccharide.

## Data Availability

The data presented in this study are available from the corresponding author upon request.

## Supplementary Materials

The following are available online at https://www.mdpi.com/article/10.3390/polym13203550/s1, Figure S1: Acrylamide monomer. Figure S2: Acrylate monomer. Figure S3: Acrylamido-2-methyl-1-propanesulfonic monomer. Figure S4: Ethylene oxide monomer. Figure S5: Guar gum monomer. Table S1: Partial charges for PAM (polyacryl amide) polymer. Table S2: Partial charges for HPAM (hydrolyzed polyacryl amide) polymer. Table S3: Partial charges for PAMPS (Acrylamido-2-methyl-1-propanesulfonic) polymer. Table S4: Partial charges for PAA (polyacrylic acid) polymer. Table S5: Partial charges for PEO (polyethylene oxide) polymer. Table S6: Partial charges for GUM (guar gum polysaccharide) polymer. Table S7: Atom type.

## References

[1] Wong S., Teng T., Ahmad A.L., Zuhairi A., Najafpour G. (2006). Treatment of pulp and paper mill wastewater by polyacrylamide (PAM) in polymer induced flocculation. J. Hazard. Mater..

[2] Moghaddam S.S., Moghaddam M.A., Arami M. (2010). Coagulation/flocculation process for dye removal using sludge from water treatment plant: Optimization through response surface methodology. J. Hazard. Mater..

[3] Renault F., Sancey B., Badot P.-M., Crini G. (2009). Chitosan for coagulation/flocculation processes—An eco-friendly approach. Eur. Polym. J..

[4] Grabsch A., Fawell P., Adkins S., Beveridge A. (2013). The impact of achieving a higher aggregate density on polymer-bridging flocculation. Int. J. Miner. Process..

[5] Tanguay M., Fawell P., Adkins S. (2014). Modelling the impact of two different flocculants on the performance of a thickener feedwell. Appl. Math. Model..

[6] Costine A., Cox J., Travaglini S., Lubansky A., Fawell P., Misslitz H. (2018). Variations in the molecular weight response of anionic polyacrylamides under different flocculation conditions. Chem. Eng. Sci..

[7] Yousefi S.A., Nasser M.S., Hussein I.A., Benamor A., El-Naas M. (2020). Influence of polyelectrolyte structure and type on the degree of flocculation and rheological behavior of industrial MBR sludge. Sep. Purif. Technol..

[8] Yousefi S.A., Nasser M.S., Hussein I.A., Judd S. (2019). Influence of polyelectrolyte architecture on the electrokinetics and dewaterability of industrial membrane bioreactor activated sludge. J. Environ. Manag..

[9] Shaikh S.M., Nasser M., Hussein I.A., Benamor A. (2017). Investigation of the effect of polyelectrolyte structure and type on the electrokinetics and flocculation behavior of bentonite dispersions. Chem. Eng. J..

[10] Shaikh S., Nasser M.S., Hussein I., Benamor A., Onaizi S.A., Qiblawey H. (2017). Influence of polyelectrolytes and other polymer complexes on the flocculation and rheological behaviors of clay minerals: A comprehensive review. Sep. Purif. Technol..

[11] Antunes E., Garcia F., Ferreira P., Blanco A., Negro C., Rasteiro M. (2010). Modelling PCC flocculation by bridging mechanism using population balances: Effect of polymer characteristics on flocculation. Chem. Eng. Sci..

[12] Nasser M., James A. (2007). Effect of polyacrylamide polymers on floc size and rheological behaviour of kaolinite suspensions. Colloids Surf. A Physicochem. Eng. Asp..

[13] Zhou Y., Franks G. (2006). Flocculation mechanism induced by cationic polymers investigated by light scattering. Langmuir.

[14] Zhou Y., Yu H., Wanless E.J., Jameson G.J., Franks G.V. (2009). Influence of polymer charge on the shear yield stress of silica aggregated with adsorbed cationic polymers. J. Colloid Interface Sci..

[15] Reis L.G., Oliveira R.S., Palhares T.N., Spinelli L.S., Lucas E.F., Vedoy D.R., Asare E., Soares J.B. (2016). Using acrylamide/propylene oxide copolymers to dewater and densify mature fine tailings. Miner. Eng..

[16] Moody G.M. (2007). Polymeric flocculants. Handbook of Industrial Water Soluble Polymers.

[17] Peng F.F., Di P. (1994). Effect of multivalent salts—Calcium and aluminum on the flocculation of kaolin suspension with anionic polyacrylamide. J. Colloid Interface Sci..

[18] Quezada G.R., Jeldres R.I., Fawell P.D., Toledo P.G. (2018). Use of molecular dynamics to study the conformation of an anionic polyelectrolyte in saline medium and its adsorption on a quartz surface. Miner. Eng..

[19] Quezada G.R., Jeldres M., Toro N., Robles P., Toledo P.G., Jeldres R.I. (2021). Understanding the flocculation mechanism of quartz and kaolinite with polyacrylamide in seawater: A molecular dynamics approach. Colloids Surf. A Physicochem. Eng. Asp..

[20] Park H.-S., Lim S., Yang J., Kwak C., Kim J., Choi S.S., Bin Kim C., Lee J. (2020). A systematic investigation on the properties of silica nanoparticles “multipoint”-grafted with poly(2-acrylamido-2-methylpropanesulfonate-co-acrylic acid) in extreme salinity brines and brine-oil interfaces. Langmuir.

[21] Xue Z., Foster E., Wang Y., Nayak S., Cheng V., Ngo V.W., Pennell K.D., Bielawski C.W., Johnston K.P. (2014). Effect of grafted copolymer composition on iron oxide nanoparticle stability and transport in porous media at high salinity. Energy Fuels.

[22] Kalaska B., Kaminski K., Miklosz J., Nakai K., Yusa S.-I., Pawlak D., Nowakowska M., Mogielnicki A., Szczubiałka K. (2018). Anticoagulant properties of poly(sodium 2-(acrylamido)-2-methylpropanesulfonate)-based di- and triblock polymers. Biomacromolecules.

[23] Zhong C., Luo P., Ye Z., Chen H. (2009). Characterization and solution properties of a novel water-soluble terpolymer for enhanced oil recovery. Polym. Bull..

[24] Hou D., Yu J., Wang P. (2019). Molecular dynamics modeling of the structure, dynamics, energetics and mechanical properties of cement-polymer nanocomposite. Compos. Part B Eng..

[25] Katiyar R.S., Jha P. (2017). Phase behavior of aqueous polyacrylic acid solutions using atomistic molecular dynamics simulations of model oligomers. Polymer.

[26] Patel K.H., Chockalingam R., Natarajan U. (2017). Molecular dynamic simulations study of the effect of salt valency on structure and thermodynamic solvation behaviour of anionic polyacrylate PAA in aqueous solutions. Mol. Simul..

[27] Jeldres M., Robles P., Toledo P.G., Saldaña M., Quezada L., Jeldres R.I. (2021). Improved dispersion of clay-rich tailings in seawater using sodium polyacrylate. Colloids Surf. A Physicochem. Eng. Asp..

[28] Quezada G., Piceros E., Robles P., Moraga C., Gálvez E., Nieto S., Jeldres R. (2021). Polyacrylic acid to improve flotation tailings management: Understanding the chemical interactions through molecular dynamics. Metals.

[29] Mpofu P., Addai-Mensah J., Ralston J. (2003). Investigation of the effect of polymer structure type on flocculation, rheology and dewatering behaviour of kaolinite dispersions. Int. J. Miner. Process..

[30] McFarlane A., Bremmell K., Addai-Mensah J. (2005). Optimising the dewatering behaviour of clay tailings through interfacial chemistry, orthokinetic flocculation and controlled shear. Powder Technol..

[31] Mudgil D., Barak S., Khatkar B.S. (2011). Guar gum: Processing, properties and food applications—A review. J. Food Sci. Technol..

[32] Castellón C.I., Piceros E.C., Toro N., Robles P., López-Valdivieso A., Jeldres R.I. (2020). Depression of pyrite in seawater flotation by guar gum. Metals.

[33] Ma X., Pawlik M. (2005). Effect of alkali metal cations on adsorption of guar gum onto quartz. J. Colloid Interface Sci..

[34] Mintis D., Mavrantzas V.G. (2019). Effect of pH and molecular length on the structure and dynamics of short poly(acrylic acid) in dilute solution: Detailed molecular dynamics study. J. Phys. Chem. B.

[35] Mintis D., Alexiou T., Mavrantzas V.G. (2020). Effect of pH and molecular length on the structure and dynamics of linear and short-chain branched poly(ethylene imine) in dilute solution: Scaling laws from detailed molecular dynamics simulations. J. Phys. Chem. B.

[36] Wang J., Wang W., Kollman P.A., Case D.A. (2006). Automatic atom type and bond type perception in molecular mechanical calculations. J. Mol. Graph. Model..

[37] Wang J., Wolf R.M., Caldwell J.W., Kollman P.A., Case D.A. (2004). Development and testing of a general amber force field. J. Comput. Chem..

[38] Cornell W.D., Cieplak P., Bayly C.I., Kollman P.A. (2002). Application of RESP charges to calculate conformational energies, hydrogen bond energies, and free energies of solvation. J. Am. Chem. Soc..

[39] Dupradeau F.-Y., Pigache A., Zaffran T., Savineau C., Lelong R., Grivel N., Lelong D., Rosanski W., Cieplak P. (2010). The R.E.D. tools: Advances in RESP and ESP charge derivation and force field library building. Phys. Chem. Chem. Phys..

[40] Frisch M.J., Trucks G.W., Schlegel H.B., Scuseria G.E., Robb M.A., Cheeseman J.R., Scalmani G., Barone V., Mennucci B., Petersson G.A. (2013). Gaussian 09, Revision D.01.

[41] Li P., Song L.F., Merz J.K.M. (2015). Systematic parameterization of monovalent ions employing the nonbonded model. J. Chem. Theory Comput..

[42] Berendsen H.J.C., Grigera J.R., Straatsma T.P. (1987). The missing term in effective pair potentials. J. Phys. Chem..

[43] Miyamoto S., Kollman P.A. (1992). Settle: An analytical version of the SHAKE and RATTLE algorithm for rigid water models. J. Comput. Chem..

[44] Abraham M.J., Murtola T., Schulz R., Páll S., Smith J., Hess B., Lindahl E. (2015). GROMACS: High performance molecular simulations through multi-level parallelism from laptops to supercomputers. SoftwareX.

[45] da Silva A.W.S., Vranken W.F. (2012). ACPYPE—AnteChamber python parser interface. BMC Res. Notes.

[46] Hess B., Bekker H., Berendsen H.J.C., Fraaije J.G.E.M. (1997). LINCS: A Linear constraint solver for molecular simulations. J. Comput. Chem..

[47] Hoover W.G. (1985). Canonical dynamics: Equilibrium phase-space distributions. Phys. Rev. A.

[48] Nosé S. (1984). A unified formulation of the constant temperature molecular dynamics methods. J. Chem. Phys..

[49] Parrinello M., Rahman A. (1981). Polymorphic transitions in single crystals: A new molecular dynamics method. J. Appl. Phys..

[50] Darden T., York D.M., Pedersen L.G. (1993). Particle mesh Ewald: AnN⋅log(N) method for Ewald sums in large systems. J. Chem. Phys..

[51] Chen P., Yao L., Liu Y., Luo J., Zhou G., Jiang B. (2012). Experimental and theoretical study of dilute polyacrylamide solutions: Effect of salt concentration. J. Mol. Model..

[52] Manning G.S. (1969). Limiting laws and counterion condensation in polyelectrolyte solutions I. Colligative properties. J. Chem. Phys..

[53] Quezada G.R., Saavedra J.H., Rozas R.E., Toledo P.G. (2019). Molecular dynamics simulations of the conformation and diffusion of partially hydrolyzed polyacrylamide in highly saline solutions. Chem. Eng. Sci..

[54] Donets S., Sommer J.-U. (2018). Molecular dynamics simulations of strain-induced phase transition of poly(ethylene oxide) in water. J. Phys. Chem. B.

[55] Jafari R., Sohrabi B. (2019). Local temperature versus system temperature in a simulation experiment containing water molecules. Phys. Chem. Chem. Phys..

