# Supplementary material for: Molecular Dynamics Study of the Conformation, Ion Adsorption, Diffusion, and Water Structure of Soluble Polymers in Saline Solutions
Source: Polymers (Basel). 2021 Oct 14;13(20):3550. doi: 10.3390/polym13203550 (PMC8539329; doi:10.3390/polym13203550)
Supplement: Supplementary file 1 [file polymers-13-03550-s001.zip › Polymers_Quezada et al_SI.pdf]

polymers MDPI

Supplementary Information: Molecular Dynamics Study of the Conformation, Ion Adsorption, Diffusion and Water Structure of Soluble Polymers in Saline Solutions

Gonzalo R. Quezada, Norman Toro, Jorge Saavedra, Pedro Robles, Iván Salazar, Alessandro Navarra and Ricardo I. Jeldres

In this support information document, we include the partial charges of all polymers from the study. The Tables S1–S6 are based on the index from the figures S1–S5. The table S7 define the atom type of the forcefield.

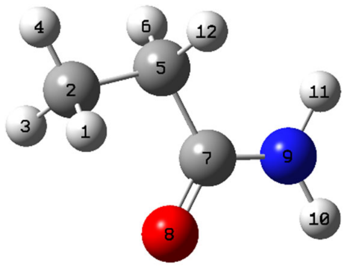

Figure S1. Acrylamide monomer.

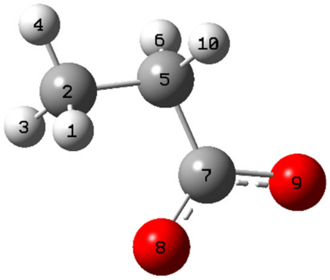

Figure S2. Acrylate monomer.

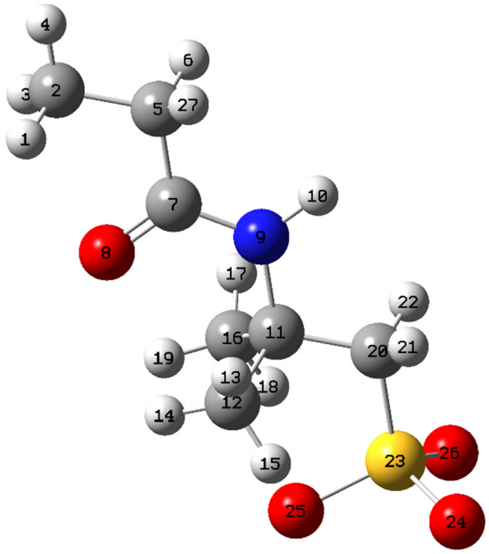

Figure S3. Acrylamido-2-methyl-1-propanesulfonic monomer.

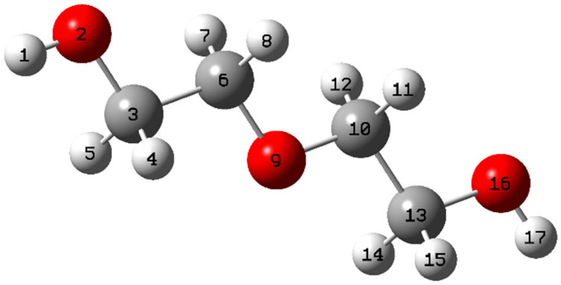

Figure S4. Ethylene oxide monomer.

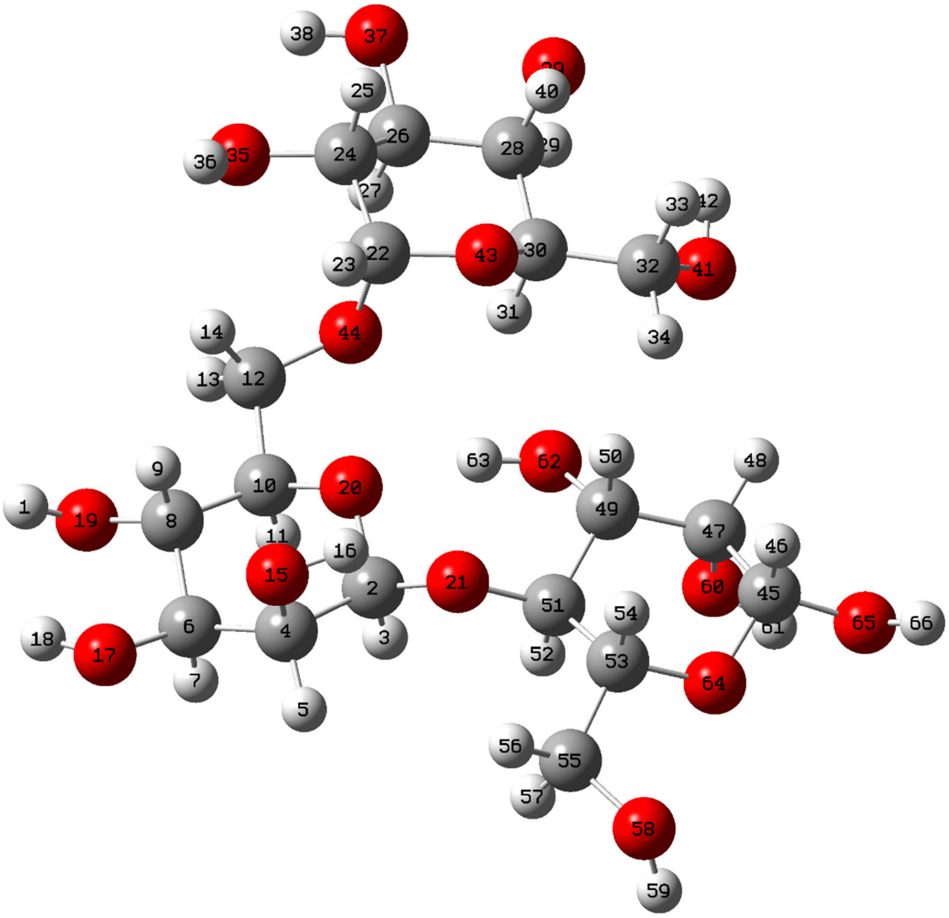

Figure S5. Guar gum monomer.

Table S1. Partial charges for PAM (polyacryl amide) polymer.

		Acrylamide Monomer		
**N°**	**GAFF**	**Initial**	**Middle**	**End**
1	hc	0.053927	-	-
2	c3	-0.196070	-0.12354	-0.020970
3	hc	0.053927	0.06116	0.024827
4	hc	0.053927	0.06116	0.024827
5	c3	-0.000670	-0.03694	-0.075570
6	hc	0.055927	0.09786	0.043527
7	c2	0.658327	0.54446	0.548827
8	o	-0.584270	-0.52464	-0.573370
9	n	-0.837470	-0.83564	-0.784070
10	hn	0.371227	0.37806	0.384227
11	hn	0.371227	0.37806	0.384227
12	hc	-	-	0.043527

Table S2. Partial charges for HPAM (hydrolyzed polyacryl amide) polymer.

		**Acrylamide Monomer**		
**N°**	**GAFF**	**Initial**	**Middle**	**End**
1	hc	0.0357364	0	-
2	c3	-0.132664	-0.11691	-
3	hc	0.035736	0.05639	-
4	hc	0.035736	0.05639	-
5	c3	0.049636	0.02119	-
6	hc	0.029436	0.11039	-
7	c2	0.600436	0.51269	-
8	o	-0.618564	-0.55311	-
9	n	-0.731564	-0.83721	-
10	hn	0.348036	0.37509	-
11	hn	0.348036	0.37509	-
12	hc	-	-	-
		**Acrylate Monomer**		
**N°**	**GAFF**	**Initial**	**Middle**	**End**
1	hc	-	-	-
2	c3	-	-0.050160	0.010522
3	hc	-	-0.017060	-0.019180
4	hc	-	-0.017060	-0.019180
5	c3	-	0.023538	0.052822
6	hc	-	0.018438	-0.063280
7	c2	-	0.528338	0.651622
8	o2	-	-0.716860	-0.770080
9	o2	-	-0.769160	-0.779980
10	hc	-	-	-0.063280

Table S3. Partial charges for PAMPS (Acrylamido-2-methyl-1-propanesulfonic) polymer.

		Acrylamide Monomer		
**N°**	**GAFF**	**Initial**	**Middle**	**End**
1	hc	0.0357364	0	-
2	c3	-0.132664	-0.11691	-
3	hc	0.035736	0.05639	-
4	hc	0.035736	0.05639	-
5	c3	0.049636	0.02119	-
6	hc	0.029436	0.11039	-
7	c2	0.600436	0.51269	-
8	o	-0.618564	-0.55311	-
9	n	-0.731564	-0.83721	-
10	hn	0.348036	0.37509	-
11	hn	0.348036	0.37509	-
12	hc	-	-	-
		**Acrylamido-2-methyl-1-propanesulfonic Monomer**		
**N°**	**GAFF**	**Initial**	**Middle**	**End**
1	hc	-	-	-
2	c3	-	-0.142250	-0.05840
3	hc	-	0.054548	0.039996
4	hc	-	0.054548	0.039996
5	c3	-	0.037048	-0.11380
6	hc	-	0.032148	0.059296
7	c2	-	0.231348	0.412596
8	o	-	-0.473750	-0.5044
9	n	-	0.004148	-0.4583
10	hn	-	0.012748	0.264696
11	c3	-	0.152748	0.297896
12	c3	-	-0.154950	-0.21540
13	hc	-	0.040848	0.051896
14	hc	-	0.040848	0.051896
15	hc	-	0.040848	0.051896
16	c3	-	-0.217250	-0.10250
17	hc	-	0.049048	0.022496
18	hc	-	0.049048	0.022496
19	hc	-	0.049048	0.022496
20	c3	-	-0.003650	-0.01090
21	h1	-	0.036948	0.029296
22	h1	-	0.036948	0.029296
23	s6	-	0.903748	0.936896
24	o3	-	-0.611590	-0.64290
25	o3	-	-0.611590	-0.64290
26	o3	-	-0.611590	-0.64290
27	hc	-	-	0.059296

Table S4. Partial charges for PAA (polyacrylic acid) polymer.

		Acrylamide Monomer		
**N°**	**GAFF**	**Initial**	**Middle**	**End**
1	hc	0.0357364	0	-
2	c3	-0.132664	-0.11691	-
3	hc	0.035736	0.05639	-
4	hc	0.035736	0.05639	-
5	c3	0.049636	0.02119	-
6	hc	0.029436	0.11039	-
7	c2	0.600436	0.51269	-
8	o	-0.618564	-0.55311	-
9	n	-0.731564	-0.83721	-
10	hn	0.348036	0.37509	-
11	hn	0.348036	0.37509	-
12	hc	-	-	-

Table S5. Partial charges for PEO (polyethylene oxide) polymer.

		Ethylene oxide Monomer		
**N°**	**GAFF**	**Initial**	**Middle**	**End**
1	ho	0.430233	-	-
2	os	-0.674770	-0.46604	-0.458390
3	c3	0.171333	0.174257	0.215506
4	h1	0.021833	0.024357	0.015906
5	h1	0.021833	0.024357	0.015906
6	c3	0.233133	0.200957	0.169506
7	h1	0.028633	0.021057	0.017706
8	h1	0.028633	0.021057	0.017706
9	os	-0.479070	-0.46604	-0.48349
10	c3	0.167633	0.174257	0.237206
11	h1	0.020533	0.024357	0.025006
12	h1	0.020533	0.024357	0.025006
13	c3	0.210833	0.200957	0.176206
14	h1	0.019333	0.021057	0.018106
15	h1	0.019333	0.021057	0.018106
16	oh	-	-	-0.67809
17	ho	-	-	0.428106

Table S6. Partial charges for GUM (guar gum polysaccharide) polymer.

		guar gum Monomer		
**N°**	**GAFF**	**Initial**	**Middle**	**End**
1	ho	0.410488	-	-
2	c3	-0.12431	-0.02373	-0.06825
3	h2	0.142488	0.144875	0.153648
4	c3	0.113688	0.050775	0.073148
5	h1	0.107588	0.121575	0.109348
6	c3	0.072688	0.053875	0.043348
7	h1	0.099188	0.101475	0.113548
8	c3	0.125288	0.002375	-0.01775
9	h1	0.110288	0.171675	0.170448
10	c3	-0.009710	-0.016330	-0.05135
11	h1	0.128188	0.117775	0.113048
12	c3	-0.045510	-0.080330	0.006448
13	h1	0.087488	0.090775	0.099348
14	h1	0.087488	0.090775	0.099348
15	oh	-0.538410	-0.533930	-0.51665
16	ho	0.390988	0.405175	0.386948
17	oh	-0.558210	-0.521330	-0.51745
18	ho	0.390688	0.365675	0.351648
19	oh	-0.592710	-0.233930	-0.20525
20	os	-0.186910	-0.193530	-0.18075
21	os	-0.220210	-0.261030	-0.24005
22	c3	0.054388	0.161775	0.112748
23	h2	0.145888	0.113175	0.152848
24	c3	0.041488	0.069675	0.126748
25	h1	0.119988	0.075675	0.105648
26	c3	0.094888	0.087275	0.092848
27	h1	0.118788	0.073275	0.071348
28	c3	0.039188	0.120675	0.061548
29	h1	0.103988	0.137075	0.131548
30	c3	0.036788	0.011875	-0.00365
31	h1	0.162588	0.054575	0.106348
32	c3	-0.054310	0.031175	0.073748
33	h1	0.108688	0.090975	0.083448
34	h1	0.108688	0.090975	0.083448
35	oh	-0.605410	-0.531930	-0.61905
36	ho	0.424988	0.331875	0.403748
37	oh	-0.606010	-0.598130	-0.59705
38	ho	0.429288	0.412975	0.401548
39	oh	-0.507510	-0.562430	-0.57485
40	ho	0.391088	0.416875	0.420048
41	oh	-0.472710	-0.574030	-0.60865
42	ho	0.346988	0.403975	0.409548

Table S6. Cont.

N°	GAFF	Acrylamide Monomer		
		**Initial**	**Middle**	**End**
43	os	-0.34701	-0.39553	-0.40995
44	os	-0.18771	-0.10103	-0.21825
45	c3	-0.02581	-0.13353	0.140948
46	h2	0.109988	0.131975	0.094048
47	c3	0.006188	-0.04473	0.062048
48	h1	0.108488	0.189775	0.116148
49	c3	0.102988	0.124675	-0.00345
50	h1	0.081888	0.103675	0.105948
51	c3	0.000787	-0.01603	-0.01845
52	h1	0.162688	0.156475	0.155948
53	c3	-0.04491	-0.02573	0.009648
54	h1	0.114088	0.113575	0.103248
55	c3	0.113288	0.117175	0.102948
56	h1	0.066188	0.065675	0.076648
57	h1	0.066188	0.065675	0.076648
58	oh	-0.59591	-0.59513	-0.60495
59	ho	0.387988	0.380675	0.391048
60	oh	-0.54341	-0.53463	-0.53145
61	ho	0.406388	0.398975	0.407248
62	oh	-0.47311	-0.47653	-0.49895
63	ho	0.287888	0.292975	0.383448
64	os	-0.14941	-0.08853	-0.27275
65	oh	-	-	-0.57525
66	ho	-	-	0.430848

Table S7. Atom type.

Atom type	Definition
c2	Sp2 aliphatic carbon
c3	Sp3 aliphatic carbon
ho	Hydroxyl hydrogen
hn	Amine/amide hydrogen
h1	Aliphatic hydrogen with 1 electro withdrawing group
h2	Aliphatic hydrogen with 2 electro withdrawing group
hc	Aliphatic hydrogen
o	Carbonyl oxygen
o2	Carboxylate oxygen
o3	Sulfonate oxygen
os	Bridging oxygen
oh	Hydroxide oxygen
n	Amine/amide nitrogen
s6	Sulfonate sulfur
